# Role of the stage-regulated nucleoside transporter *Tb*NT10 in differentiation and adenosine uptake in *Trypanosoma brucei*

**DOI:** 10.1016/j.molbiopara.2007.04.006

**Published:** 2007-07

**Authors:** Iris Spoerri, Ruth Chadwick, Christina Kunz Renggli, Keith Matthews, Isabel Roditi, Gabriela Burkard

**Affiliations:** a Institute of Cell Biology, University of Bern, Baltzerstrasse 4, 3012 Bern, Switzerland; b Institute of Immunology and Infection Research, School of Biological Sciences, University of Edinburgh, West Mains Road, Edinburgh EH9 3JT, UK; c Swiss Tropical Institute Basel, Socinstrasse 57, 4002 Basel, Switzerland

**Keywords:** UTR, untranslated region, SIF, stumpy inducing factor, GFP, green fluorescent protein, *Tb*NT10, nucleoside transporter 10 of *Trypanosoma brucei*, ORF, open reading frame, IC, inhibitory concentration, Adenosine, Nucleoside transporter, Differentiation, Stumpy inducing factor, Trypanosomes

*Trypanosoma brucei* undergoes complex metabolic and morphological changes during its life cycle in order to adapt and survive inside the mammalian host and the tsetse fly vector. One of these adaptations to different environments is reflected by the expression of various nucleoside transporters. As protozoan parasites are unable to synthesise the purine ring *de novo*, they have to salvage preformed nucleosides and nucleobases from their hosts [1]. To date, several nucleoside transporters of *T. brucei* have been identified and characterised, all of which belong to the equilibrative nucleoside transporter family. Depending on their substrate specificities they can be assigned to two types of transport activities, P1 and P2 [2]. P1 activity is specific for the uptake of adenosine, guanosine and inosine and has been found in both bloodstream and procyclic forms. P2 activity is specific for adenosine and adenine uptake. Only one P2 transporter, *Tb*AT1, has been described so far. *Tb*AT1 is expressed in bloodstream forms and has been shown to transport the trypanocidal drugs melarsoprol and pentamidine [2,3]. Additionally, purines can be salvaged by the hypoxanthine transporting activities H1–H4 [4,5].

Although many nucleoside transporters and their substrate specificities have been characterised, the reason for the parasite to employ many related transporters with the seemingly redundant function of purine uptake, is still unclear. Furthermore, very little is known about the contribution of single transporters to purine uptake in different life cycle stages of the parasite.

The nucleoside transporter *Tb*NT10 [6] (Tb09.160.5480, also known as *Tb*AT-B [7]), displays a P1-type transport activity with high affinities for adenosine, guanosine and inosine and marginal affinities for hypoxanthine and adenine [6,7]. *TbNT10* mRNA was shown to be stage-regulated in *T. brucei rhodesiense EATRO* 2340*,* with highest expression in short stumpy bloodstream forms and lowest expression in long slender bloodstream and procyclic forms [6]. This regulated expression during the life cycle might point to a function of the transporter in the process of differentiation.

In this study a possible role of *Tb*NT10 in differentiation from long slender to short stumpy bloodstream forms, and in the following differentiation step to procyclic forms, was investigated. Northern blot analysis with mRNA from different life cycle stages of *T. brucei brucei* strain AnTat 1.1 [8,9] confirmed that the *TbNT10* mRNA is expressed at a low level in long slender bloodstream forms and is up-regulated in short stumpy bloodstream forms (Fig. 1A). In this strain, however, the highest level of *TbNT10* mRNA was found in procyclic forms. Strain-specific differences have been observed previously for the expression of another P1-type transporter *Tb*NT2 [10]. In addition, culture conditions might influence expression levels of certain transporters. The *TbNT10* mRNA is 4.2 kb long, with an open reading frame (ORF) of 1.4 kb. In order to determine the length of the untranslated regions (UTRs) a cDNA library [11] was screened and cDNAs containing spliced leader sequences were analysed. The 5′ UTR was found to be 42 bases. We could not determine the precise length of the 3′ UTR, as this region contains several AU-rich stretches that supported internal priming. Considering the Northern blot data, however, we estimate the 3′ UTR to be ∼2.7 kb long.

As mentioned above, the regulated expression of *TbNT10* mRNA could point to several functions in the differentiation from long slender to short stumpy bloodstream forms. Short stumpy forms are pre-adapted to be taken up by the tsetse fly. They are unable to divide in the mammalian bloodstream and die within a few days. The differentiation from long slender into short stumpy bloodstream forms is triggered by a stumpy inducing factor (SIF) that accumulates in a cell-density dependent manner in culture medium (and presumably blood) containing long slender parasites [12]. SIF has been characterised as a small compound with a molecular weight of ≤500 Da. Its chemical identity is still unclear, but it could potentially be a nucleoside since membrane permeable cAMP analogues mimic SIF activity [12].

In order to investigate the role of *Tb*NT10 in the response to SIF, a null mutant (*Δnt10::NEO*/*Δnt10::HYG*) was constructed in *T. brucei* AnTat 1.1 (Fig. 1A). For technical reasons, this was done in procyclic forms, as these are considerably easier to transfect. Southern blot analysis confirmed the correct integration of the two antibiotic resistance cassettes into the *TbNT10* loci (data not shown) and Northern blot analysis confirmed the absence of *TbNT10* mRNA in the null mutant (Fig. 1A). Early procyclic forms of the null mutant showed no phenotype in growth or in differentiation from early to late procyclic forms, as assessed by the loss of GPEET procyclin [13] (data not shown). Furthermore, the null mutant was still able to infect tsetse flies (*Glossina morsitans morsitans*), and subsequently mice, from which long slender bloodstream forms were isolated for further analysis. In order to test whether *Tb*NT10 is required for differentiation from the long slender to short stumpy bloodstream form, cultures of the wild type and the *Tb*NT10 null mutant were compared (Fig. 1B). Differentiation to the stumpy form was monitored by the diaphorase assay [14]. The null mutant entered stationary phase at a lower cell density, but differentiated to the short stumpy bloodstream form as fast as the wild type. To determine if SIF might be taken up by other P1 transporters, wild-type cells were cultured in the presence of 1 mM inosine, which is a P1 substrate and, hypothetically, could compete with SIF (Fig. 1B). However, wild-type cells exposed to inosine were able to differentiate from long slender to short stumpy bloodstream forms as well as cells without the competitor. These results indicate that neither *Tb*NT10, nor any other P1 transporter, is responsible for the uptake of SIF in cell culture. In addition, a function of *Tb*NT10 in the maintenance of short stumpy forms or the differentiation to procyclic forms could be excluded, as stumpy forms of the null mutant were still able to differentiate to procyclic forms and express EP procyclins with identical kinetics to the wild type (data not shown).

The situation *in vivo* was analysed by comparing mice infected with the *Tb*NT10 null mutant and the wild type. Total parasitaemia, the proportion of dividing and non-dividing cells and differentiation from long slender to short stumpy bloodstream forms were compared between the two groups (Fig. 1C, D). The null mutant parasites established a peak parasitaemia similar to the wild type, but showed a two-fold lower cell density 6–10 days post infection. However, the ratio between dividing and non-dividing cells (Fig. 1C) and the kinetics of differentiation into growth-arrested short stumpy bloodstream forms (Fig. 1D) were the same as for wild-type cells. These data confirmed the findings *in vitro* that *Tb*NT10 is not involved in the differentiation of long slender to short stumpy bloodstream forms. The lower parasite density during differentiation to short stumpy bloodstream forms *in vivo* as well as *in vitro* might point to a shortage of purines in the *Tb*NT10 null mutant. Nevertheless, *Tb*NT10 is not an essential gene, as the null mutant could be transmitted by tsetse flies and complete the entire life cycle.

An ectopically expressed GFP-*Tb*NT10 fusion protein showed a surface localisation in procyclic forms (Fig. 2A). Taken together with the high expression of *TbNT10* mRNA in early and late procyclic forms (Fig. 1A), this points to a likely function for the transporter in the insect stages of the parasite, with the up-regulation of mRNA in stumpy forms perhaps being a pre-adaptation for this. So far, two P1-type nucleoside transport activities *Tb*NT2 and *Tb*NT5 [10] have been described for procyclic forms and *Tb*NT10 might also contribute to the uptake of purines and purine analogues. As a first step we measured the sensitivity to two toxic adenosine analogues, tubercidin (7-deazaadenosine) and cordycepin (3′-deoxyadenosine). The IC_50_ values of procyclic forms for tubercidin and cordycepin were found to be in the μM range, which is approximately 1000-fold higher than described for bloodstream forms [15]. In the drug sensitivity assays, procyclic forms of the null mutant were found to be 3.6 times more resistant to both tubercidin and cordycepin compared to wild-type parasites (Fig. 2C). In contrast, there was no difference between the wild type and *Tb*NT10 null mutant in their sensitivity to the two trypanocidal drugs, that are taken up by the P2 transporter *Tb*AT1 in bloodstream forms [2,16], melarsen oxide and pentamidine (data not shown). To confirm that the increased resistance to tubercidin and cordycepin was due to the depletion of *Tb*NT10, two independent addback clones were constructed (Fig. 2B and C). The *TbNT10* mRNA levels in both clones were comparable to that of the endogenous transcript, as shown by Northern blot (Fig. 2B). Ectopic expression of *Tb*NT10 not only restored the sensitivity to tubercidin, but enhanced it up to 13-fold compared to wild-type cells. A similar trend was found in the response to cordycepin, as the two addback clones became approximately five-fold more sensitive than wild-type cells. In order to determine the contribution of *Tb*NT10 to total P1-type purine uptake, two substrates, inosine and adenosine, were added to the drug assays as competitors (Fig. 2C). An excess of competitor (1 mM) increased drug resistance in all cell types, except the null mutant. The addition of competitors did not substantially increase the resistance of the wild type above that of the null mutant (without competitors); however, indicating that most P1-type transport was due to *Tb*NT10.

Although the addbacks became more resistant to tubercidin in the presence of either competitor, they did not reach the same level of resistance as the wild type, possibly because the transporters were not saturated. Unexpectedly, however, they became highly resistant to cordycepin in the presence of adenosine. This could reflect the mode of action of cordycepin which, unlike tubercidin, is able to compete with adenosine intracellularly for integration into nucleic acids. Alternatively, this could be a consequence of different affinities of *Tb*NT10 for the various substrates. Another unexpected finding was that the null mutant became more sensitive to cordycepin when competitors, particularly adenosine, were added. A similar outcome was recently reported for a *Tb*AT1 null mutant in bloodstream forms that became hypersensitive to cordycepin in the presence of adenine [15]. One possibility is that crosstalk between different transporters might be involved in this effect, as other transporters could be up-regulated in the null mutant and might also contribute to drug uptake. Thus, although we cannot explain all effects observed in the drug assays, they clearly show a significant contribution by *Tb*NT10 in the uptake of purine analogues by procyclic forms of *T. brucei.*

In order to quantify the contribution of *Tb*NT10, the rates of adenosine uptake were compared in the wild type, the null mutant and one addback clone (Fig. 2D). Wild type cells showed strong adenosine uptake in the range of 50 pmol/10^7^ cells after 5 min. Consistent with the results from the drug sensitivity assays, adenosine uptake by the addback mutant was increased to 70 pmol/10^7^ cells, while uptake by the *Tb*NT10 null mutant never exceeded 10 pmol/10^7^ cells after 5 min (Fig. 2D). Once again, these results indicate a major role for *Tb*NT10 in adenosine uptake by procyclic forms, although the parasites clearly have other means to acquire purines (for example, via hypoxathine transporters).

In conclusion, the stage regulated nucleoside transporter *Tb*NT10 is not involved in the differentiation from long slender to short stumpy forms or in the transition to procyclic forms. It is not an essential gene, since null mutants can complete the whole life cycle. Nevertheless, we have demonstrated that in procyclic forms, where high levels of *TbNT10* mRNA are expressed, purines and toxic analogues are imported by *Tb*NT10 and that it is the main transporter for adenosine in this life cycle stage.

## Figures and Tables

**Fig. 1:**
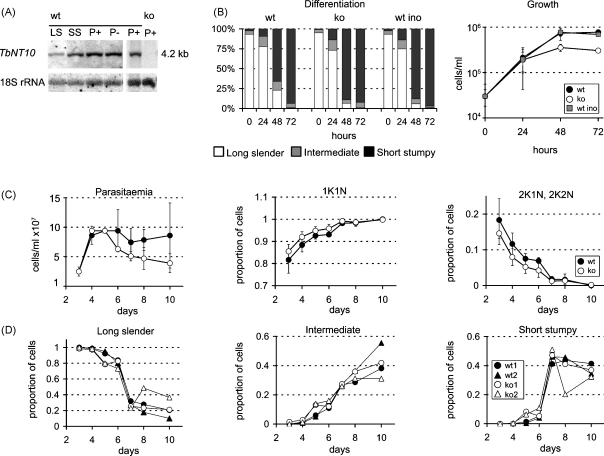
*TbNT10* mRNA expression and role in differentiation. (A) Northern blot analysis with 10 μg total RNA from long slender (LS) and short stumpy (SS) bloodstream forms, and early (P+) and late (P−) procyclic forms of AnTat 1.1 wild type (wt) and the *Tb*NT10 null mutant (ko). The complete ORF of *Tb*NT10 was amplified from genomic DNA by PCR with the primers *NT10*-orf1 5′-ATGCTCGGGTTTGAGTCGTT-3′ and *NT10*-orf2 5′-TTATGATTCTGGAAGAGCCG-3′ and used as template to synthesise a ^32^P-labelled probe for hybridisation. The size of the *TbNT10* mRNA reported in the original publication [6] was incorrect, this being due to a misunderstanding between the two laboratories involved. The correct size of the transcript has been independently confirmed as being ∼4.2 kb (K. Matthews, unpublished data). The signals were normalised using a γ^32^P-dATP end-labelled probe specific for 18S rRNA [17]. The *Tb*NT10 null mutant was constructed in early procyclic forms of *Trypanosoma brucei* AnTat 1.1 by sequentially replacing the ORFs by a neomycin- and a hygromycin-resistance cassette, respectively. The 5′- and 3′-flanking regions of the ORF were amplified by PCR using the primers: *NT10*-*Kpn*I 5′-CAGGTACCAAACTGACGAAAGTGC-3′, *NT10*-*Hin*dIII 5′-GGAAGCTTCTTGCTTAAATGACTCAG-3′, *NT10*-*Bam*HI 5′-TTGGATCCCTAAGAGGAGGTAA-3′ and *NT10*-*Xba*I 5′-TATCTAGACACATTTGTGGGCGCG-3′. The products were cloned into pBluescript upstream and downstream of a neomycin- or a hygromycin-resistance gene, respectively, using the restriction sites (underlined) introduced by the primers. Stable transformation was performed as described [18]. Inserts were excised by digestion with *Kpn*I and *Xba*I prior to electroporation. (B) *In vitro* differentiation from long slender to short stumpy bloodstream forms. Long slender bloodstream forms of the *Tb*NT10 null mutant and the wild type were thawed from frozen mouse blood stabilates and cultured with a starting density of 3 × 10^4^ cells/ml in HMI-9 medium containing 0.65% low melting temperature agarose and 10% horse serum. The 1 mM inosine was added to wild-type cells where indicated (wt ino). After 0, 24, 48, and 72 h of incubation at 37 °C and 5% CO_2_ the percentage of long slender, intermediate and short stumpy forms was determined by the diaphorase assay [14]. A representative experiment (from four independent experiments with similar results) is shown. Growth of the cells was monitored during differentiation and the means of three experiments ± standard deviation (S.D.) are shown. (C) *In vivo* differentiation from long slender to short stumpy bloodstream forms. Four MF1 mice were infected with bloodstream forms of wild type or *Tb*NT10 null mutant parasites, respectively. Parasitaemia was measured every 24 h from day 3 until day 10 post infection and the means ± S.D. are shown (two out of four mice infected with wild-type parasites died on day 9 of the experiment). At the same time points, cell division was monitored by DAPI staining and microscopic analysis of the numbers of nuclei (N) and kinetoplasts (K) per cell. The proportion of cells with 1K1N (comprising cells arrested in G1/G0 as well as proliferative cells early in their cell cycle) and dividing (2K1N, 2K2N) cells are shown. (D) The *in vivo* differentiation of the parasites described above was monitored by microscopic analysis of the morphology; 250 cells were analysed per sample and categorised as long slender, intermediate or short stumpy bloodstream forms. Data points represent the parasite populations in individual mice infected with the wild type (wt1, wt2) and the null mutant (ko1, ko2).

**Fig. 2:**
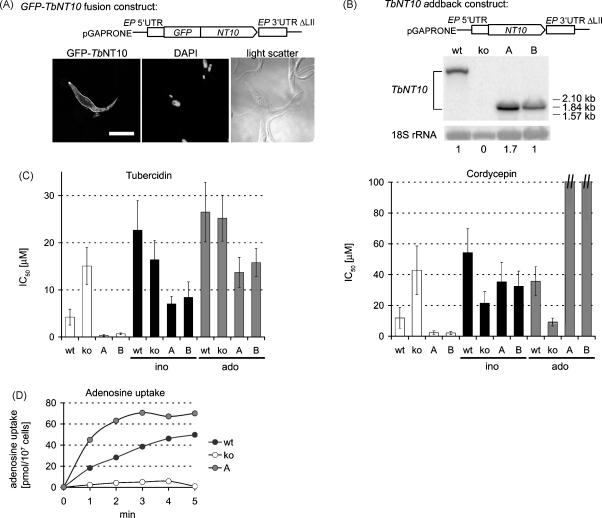
(A) Localisation of a GFP-*Tb*NT10 fusion protein. The *TbNT10* ORF was cloned into the *Bam*HI site of the vector pG-EGFP-ΔLIIγ [18] downstream of the *GFP* ORF. The construct was transiently transfected into AnTat 1.1 procyclic forms and expression of the GFP-*Tb*NT10 fusion protein was analysed after 24 h by confocal microscopy. Scale bar indicates 10 μm. (B) Construction of *Tb*NT10 addback mutants. The ORF of *TbNT10* was cloned into the *Eco*RI site of pGAPRONE-ΔLII containing a puromycin resistance cassette [19]. The insert was excised using *Kpn*I and *Not*I and stably transfected into early procyclic forms of the *Tb*NT10 null mutant. Two independent addback clones were analysed for correct integration into an EP procyclin locus. About 10 μg total mRNA of the wild type (wt), the null mutant (ko) and the two addback clones (A and B) were subjected to Northern blot analysis as described above. The relative amounts of *TbNT10* mRNA are indicated under each lane. (C) Sensitivity to toxic adenosine analogues. IC_50_ values of the wild type, the null mutant and the addback clones for tubercidin and cordycepin were determined by Alamar blue assays [15]. The assays were carried out without competitor (empty bars) or in the presence of 1 mM inosine (ino) or 1 mM adenosine (ado), respectively, as indicated (filled bars). The means ± S.D. from at least five experiments are shown for each cell line. (D) Uptake assays with ^3^H-labelled adenosine were performed with wild type, null mutant and addback clone A, as described [20]. One out of eight experiments with comparable results is shown.
